# Did a quality improvement intervention improve quality of maternal health care? Implementation evaluation from a cluster-randomized controlled study

**DOI:** 10.1093/intqhc/mzz126

**Published:** 2019-12-12

**Authors:** Elysia Larson, Godfrey M Mbaruku, Jessica Cohen, Margaret E Kruk

**Affiliations:** 1 Department of Global Health and Population, Harvard T.H. Chan School of Public Health, Boston, USA; 2 Department of Biostatistics, Harvard T.H. Chan School of Public Health, Boston, MA, USA; 3 Ifakara Health Institute, Dar es Salaam, Tanzania

**Keywords:** quality improvement, maternal health, cluster-randomized controlled study, Tanzania, quality measurement, implementation science

## Abstract

**Objective:**

To test the success of a maternal healthcare quality improvement intervention in actually improving quality.

**Design:**

Cluster-randomized controlled study with implementation evaluation; we randomized 12 primary care facilities to receive a quality improvement intervention, while 12 facilities served as controls.

**Setting:**

Four districts in rural Tanzania.

**Participants:**

Health facilities (24), providers (70 at baseline; 119 at endline) and patients (784 at baseline; 886 at endline).

**Interventions:**

In-service training, mentorship and supportive supervision and infrastructure support.

**Main outcome measures:**

We measured fidelity with indictors of quality and compared quality between intervention and control facilities using difference-in-differences analysis.

**Results:**

Quality of care was low at baseline: the average provider knowledge test score was 46.1% (range: 0–75%) and only 47.9% of women were very satisfied with delivery care. The intervention was associated with an increase in newborn counseling (β: 0.74, 95% CI: 0.13, 1.35) but no evidence of change across 17 additional indicators of quality. On average, facilities reached 39% implementation. Comparing facilities with the highest implementation of the intervention to control facilities again showed improvement on only one of the 18 quality indicators.

**Conclusions:**

A multi-faceted quality improvement intervention resulted in no meaningful improvement in quality. Evidence suggests this is due to both failure to sustain a high-level of implementation and failure in theory: quality improvement interventions targeted at the clinic-level in primary care clinics with weak starting quality, including poor infrastructure and low provider competence, may not be effective.

## Introduction

Recent trends in increased use of facilities for childbirth have not always been accompanied by declines in maternal and newborn mortality [2, 3], which remain unacceptably high [4, 5]. Part of the gap between facility use and reduced mortality is likely due to poor quality of care.

The World Health Organization recommends that most deliveries occur in primary care facilities. This recommendation is based on the expectation that primary care facilities are equipped to conduct normal deliveries and can provide timely referral for complications [6, 7]. However, the quality of maternal and newborn care at primary care facilities is often low [8–10]. For example, a Tanzanian study found that in rural public primary care clinics, only 69% of providers reported implementing any oxytocic, an intervention that should occur for every delivery [11]. Despite indications of quality constraints, a substantial proportion of facility deliveries occur in primary care clinics in Tanzania [12].

Given international and local recommendations to perform deliveries at the primary care level and the evidence of gaps in quality of care, we designed an intervention that focused on directly influencing provider behavior and care delivery at the primary care level. The intervention model was motivated by successful multi-component interventions designed to improve care and treatment for HIV [13, 14] and was explicitly designed to be sustained within the health system.

While quality improvement interventions are ubiquitous in many regions with weak service delivery, rigorous evaluations of interventions in context are scarce. This paper presents an implementation evaluation of a quality improvement program on quality of obstetric care in rural Tanzania. We report implementation strength and fidelity of the program over 4 years. The results can inform quality improvement approaches in challenging health system contexts, and the methodological approach can inform future implementation science studies.

## Methods

### Study setting

This study was implemented in 24 primary care clinics, or dispensaries, in four districts of Pwani Region, Tanzania. Selection criteria were previously described in detail [15]. Dispensaries are outpatient facilities programed to provide primary care, including reproductive health services [16, 17]. In Pwani, 73% of deliveries occurred in health facilities in 2010, and around one third of those occurring in health facilities occurred in primary care facilities [12].

### Intervention

We stratified the 24 facilities by district and then randomized facilities in a 1:1 ratio to either the intervention or the control group, resulting in three intervention and three control facilities in each district. Randomization occurred by pulling facility names out of a hat in the presence of research staff and regional health officials. Clusters were defined as the health facility and the surrounding catchment area. Facilities in the intervention group received a maternal and newborn health quality improvement intervention, while facilities in the control group continued with standard care.

Delivery of interventions known to avert maternal and newborn deaths (e.g. high quality antenatal care (ANC) and rapid deployment of emergency care) [18] requires competent and motivated providers working within well-equipped facilities that are able to support basic emergency obstetric and newborn care (BEmONC), with appropriate access to referral facilities. The MNH+ intervention uses BEmONC training to provide a review of foundational knowledge, complemented by continuous mentoring and supportive supervision by an obstetrician, and provision of the necessary equipment, supplies, and medication. Our theory of change is that these quality inputs will translate into better quality process of care and outcomes (box). Implementation of the intervention began in June 2012; by July 2013, the full intervention was underway and continued into the spring of 2016.

**Box:** Theory of change and intervention components

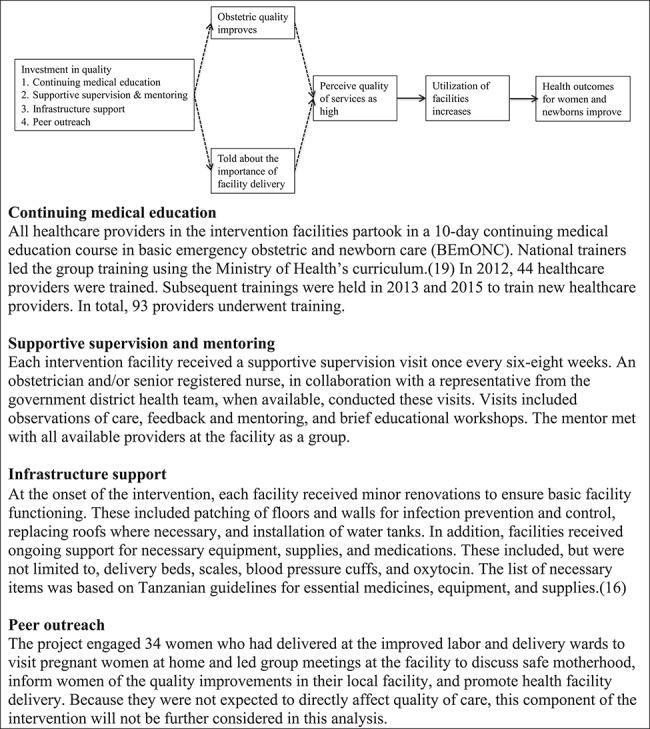

### Study measures

#### Implementation index

We developed an implementation index to assess the effect of variation of the intervention across the 12 intervention facilities [20, 21]. For each intervention component, we identified indicators for the dose delivered (e.g. proportion of expected supportive supervision visits delivered), reach to the intended audience (e.g. proportion of providers who are trained) and dose received (e.g. provider’s training scores).

#### Fidelity: quality of care

Fidelity is defined as the correct application of the program [21]. Instead of looking at whether each individual intervention component was implemented as intended, we chose a more demanding definition of fidelity: whether the immediate intended effect, that is improvement in quality, was achieved. We thus specified a range of quality metrics using Donabedian’s model of quality of care of structure, process, and outcome.

#### Quality: structure

Trained providers completed a 60-question multiple-choice test that emphasized obstetric and newborn emergency care and two clinical vignettes that tested their clinical judgment in obstetric emergencies (appendix 1), receiving a continuous score between 0 and 1 on each instrument.

#### Quality: process

We used data from facility registers to create a composite indicator of routine obstetric services (appendix 2). For each facility, we created an indicator for the sum of each of the six BEmONC signal functions (life-saving health services) that had been performed in the previous 3 months. We measured reported receipt of services as the proportion of women receiving a uterotonic, the proportion of women receiving IV antibiotics and a composite indicator of counseling on six items. We measured patients’ perception of quality through composite indicators for nontechnical quality and technical quality.

#### Quality: outcomes

We asked patients and providers to report their perception of quality at the facility. Patients also reported their satisfaction with delivery care. Indicators were created to compare those with the top rating (e.g. excellent or very satisfied) to all others.

We measured four indicators of maternal health through biomarkers collected during the household survey: lack of anemia (hemoglobin level is 12.0 g/dl or above for nonpregnant women and 11.0 g/dl or above for pregnant women [22]), lack of hypertension (average systolic reading less than 140 mm Hg and average diastolic reading less than 90 mm Hg [23]), distribution of EQ-5D (EuroQol Group, Rotterdam, Netherlands) and distribution of mid-upper arm circumference (MUAC).

### Study participants and data collection

#### Patient data (fidelity: processes and outcomes)

Patient-level data were collected as repeated cross-sections in 2012, 2014 and 2016 (Appendix 2 for summary) [15, 24, 25]. All households in the catchment area were enumerated. The sample size was determined based on another primary outcome, utilization. At midline, we selected 60% of women from each catchment area using a simple random sample. Women were eligible for the household survey if they were at least 15 years of age and lived within the catchment area of a study facility, and included in this analysis if they had delivered their most recent child between 6 weeks and 1 year prior to the interview in one of the study facilities. At midline and endline, women were invited to have their hemoglobin and blood pressure tested.

#### Healthcare provider data (fidelity: structures and processes)

The job satisfaction survey was offered to all healthcare providers [26], while the obstetric knowledge test and the clinical vignettes were offered to healthcare providers who had received formal pre-service training in obstetric care (i.e. clinical officers and nurses).

#### Healthcare facility data

The facility audit was adapted from the needs assessment developed by the Averting Maternal Death and Disability Program and the United Nations system [27]. The audit asked about services routinely provided by that facility. In addition, we collected aggregate monthly indicators of use and quality from the facility registers and partographs.

The provider surveys, facility audits and register abstraction were conducted annually.

#### Implementation index data

The implementation team at Tanzania Health Promotion Support (THPS) collected data on intervention delivery. Data collection methods are further described in appendix 2.

#### Ethical considerations

All women and healthcare providers participating in surveys provided written, informed consent prior to participation. Ethics review boards in both Tanzania, National Institute for Medical Research and Ifakara Health Institute and in the U.S., Columbia University and the Harvard T.H. Chan School of Public Health approved this study.

### Statistical analyses

Completed surveys were imported into Stata version 14.2 for cleaning and analysis. We first conducted descriptive statistics then assessed the implementation and fidelity of the intervention.

#### Implementation index

Each of the three indicators (dose delivered, dose received and reach) were multiplied together to obtain a composite indicator for each of the three components (infrastructure, training and supportive supervision) [21, 28]. These three scores were then averaged to create a single composite measure of implementation strength. Complete implementation would thus be represented by a score of ‘1’ and complete failure of implementation by a score of ‘0’.

#### Fidelity: difference-in-differences analysis of the effect of the intervention on achieved obstetric quality

To measure the effect of the MNH+ intervention on obstetric quality, we conducted difference-in-differences analyses assessing the difference between intervention and control facilities in the change of each quality indicator from baseline (2012) to endline (2016). These analyses control for both differences in quality patterns between facilities at baseline and changing patterns over time that are external to the intervention but consistent across the region. We included a fixed effect for district to account for stratification during the design phase. Except where noted, all models used generalized estimating equations with an exchangeable correlation structure. For binary quality measures, we used a log link to estimate risk ratios [29]. The robust sandwich estimator was used to account for clustering at the facility level. Because anemia and hypertension were not measured at baseline, we could not conduct a difference-in-differences analysis. Instead, we compared intervention to control at endline and adjusted for age, household wealth and district [30, 31]. Additionally, we assessed whether there was an effect of the intervention on the quality results at midline (2014).

To assess changes in provider knowledge and competence, our primary analysis evaluated within provider changes. Because of unexpectedly low retention of providers across the five-year study period, we assessed changes from baseline (2012) to first follow-up (2013). We conducted a secondary analysis to measure changes in mean facility knowledge score from baseline (2012) to endline (2016). We conducted linear regression with a fixed effect for district and the robust sandwich estimator to account for clustering at the facility level.

#### Sub-group analysis of the impact of high-implementation facilities on obstetric quality

We conducted a sub-group analysis to assess the impact of the intervention in the high-implementation facilities (top third) compared to control facilities (*N* = 12) through difference-in-differences analyses.

## Results

We interviewed 3,019 women at baseline and 3,575 women at endline, 3,146 of whom delivered 6 weeks to 1 year prior to interview. Of those women, 784 (26%) delivered in their local primary clinic at baseline and 886 (28%) at endline and were thus included in this analysis (Appendix 3). On average, women were 28 years old at baseline (Table 1). At endline, of those providers who participated in the baseline survey, 12 (32%) completed the knowledge test, 9 (26%) completed the vignettes and 30 (43%) completed the satisfaction survey. Most providers were female (Table 2, Appendix 4).

**Table 1:** Characteristics of women who participated in the baseline household survey and reported delivering their most recent child in their catchment facility, Pwani region, Tanzania (2012)

	Control (*N* = 352) **mean or percent**	Intervention (*N* = 432) **mean or percent**
Demographics		
Age (mean)	28.1	27.5
Education (categorical)		
No formal	25.6%	26.0%
Some primary	13.4%	11.4%
Completed primary	51.9%	54.3%
Any secondary	9.1%	8.4%
Farmer or homemaker	82.2%	80.2%
Muslim	83.8%	85.4%
Married or living with partner	83.8%	85.4%
Household assets		
Media index (mean)^1*^	3.47	3.35
Mobile phone	72.2%	77.0%
Electricity	8.0%	6.0%
Consumes > 2 meals per day	89.8%	89.1%
Community characteristics		
Village has paved road	28.8%	54.6%
District		
Bagamoyo	34.1%	53.7%
Kibaha Rural	11.1%	1.9%
Kisarawe	18.8%	23.4%
Mkuranga	36.1%	21.1%

**Notes**: Women were eligible for inclusion if they had delivered a child in the 6 weeks to 1 year prior to interview at their designated catchment facility. Baseline interviews took place in February–April 2012. There were 24 study facilities; catchment areas consist of villages designated by the local government.

^1^Media index derived from the frequency of reading a newspaper, listening to the radio and watching television; possible range (0, 12)

^*^Difference between intervention and control group is statistically significant at the α = 0.10 level

**Table 2:** Characteristics of study facilities and healthcare providers working in one of the 24 study facilities at baseline (2011–2012)

	Control (*N* = 35)	Intervention (*N* = 51)
**Provider characteristics**	**Mean or percent**	**Mean or percent**
Female	77.1%	74.5%
Age^*^	42.3	37.9
Cadre		
Clinical officer	34.3%	27.5%
Nurse	22.9%	19.6%
Medical attendant ^1^	40.0%	47.1%
Other	2.9%	5.9%
Full time employment	90.9%	97.3%
Worked in study facility for more than 2 years^*^	87.9%	59.5%
District of employment		
Bagamoyo	31.4%	25.5%
Kibaha Rural	28.6%	21.6%
Kisarawe	28.6%	29.4%
Mkuranga	11.4%	23.5%
**Facility characteristics**		
Workload^2^		
Number of facility deliveries	5.9	7.9
Number of outpatient visits	240.1	255.4
Number of healthcare workers at facility	3.6	4.2

**Notes:** Healthcare providers were eligible for inclusion if they were working at the study facility at the time of interview. Baseline interviews took place in December 2011 to May 2012. Full table for all years can be found in Appendix 3.

^1^Includes medical attendants and maternal and child health aides.

^2^Data represent average monthly use from January–December 2011 and are determined from the facility monthly registers

^*^Difference between intervention and control group is statistically significant at the α = 0.10 level

The average score on the implementation index was 0.39 (range: 0.26–0.53). The average scores on the dose delivered indicators for infrastructure training, and supportive supervision were 83.3, 64.6, and 67.4%, respectively. The scores for reach were 77.8, 68.9 and 83.3%, respectively, and for dose received were 100, 69.5, and 75.0%.

At endline, of women who delivered their baby in their local intervention facility, 61% reported being very satisfied with their delivery care, compared to 65% of women in control facilities (Table 3). No statistically significant improvements in measures of process of care were found, except for the receipt of newborn counseling (0.74, 95% CI: 0.13, 1.35). The results at midline were similar (Appendix 5).

**Table 3:** The effect of the MNH+ intervention on the quality of care in government-managed primary healthcare from 2012 to 2016, difference-in-differences analysis

	Control baseline mean or percent	Control follow-up mean or percent	Control diff^1^	Interv. baseline mean or percent	Interv. follow-up mean or percent	Interv. diff^2^	β or RR (95% CI)
Processes							
Provision of evidence-based care							
Routine care (3 items)^3^	1.75	1.93	0.18	1.90	2.24	0.34	0.16 (−0.03, 0.35)
Basic emergency obstetric and newborn care (6 items)^4^	2.08	2.42	0.34	2.08	2.58	0.50	0.17 (−1.16, 1.50)
Receipt of services by women							
Receipt of IV antibiotic	23.1%	22.9%	−0.2%	18.8%	16.1%	−2.7%	0.86 (0.45, 1.65)
Receipt of uterotonic	75.9%	89.7%	13.8%	82.1%	92.9%	10.8%	0.98 (0.84, 1.12)
Receipt of newborn counseling (6 items)^6^	4.49	4.46	−0.03	4.25	5.15	0.90	0.74^*^ (0.13, 1.35)
Patient experience and care competence							
vNontechnical quality (5 items)^7^	1.16	1.40	0.24	1.12	1.49	0.37	0.11 (−0.08, 0.30)
vTechnical quality (2 items)^8^	0.10	0.18	0.08	0.13	0.22	0.09	−0.03 (−0.16, 0.10)
Outcomes							
Health outcomes^9^							
Patient is not anemic	-	40.8%	-	-	36.3%	-	0.90 (0.78, 1.05)
Patient is not hypertensive	-	91.7%	-	-	90.9%	-	0.99 (0.97, 1.02)
Maternal mid-upper arm circumference	27.03	28.15	1.12	27.37	28.02	0.65	−0.44 (−0.98, 0.10)
EQ-5D	0.93	0.95	0.02	0.93	0.95	0.02	0.01 (−0.01, 0.03)
Overall quality and satisfaction1^0^							
Patient satisfaction with delivery care	47.9%	64.9%	17.0%	47.6%	60.9%	13.3%	0.95 (0.69, 1.30)
Patient perceived quality of delivery care	14.5%	19.1%	4.6%	13.0%	21.2%	8.2%	1.22 (0.58, 2.59)
Provider perceived quality of ANC	15.2%	42.6%	27.4%	27.0%	35.4%	8.4%	0.46 (0.11, 1.87)
Provider perceived quality of labor care	24.3%	35.2%	10.9%	29.7%	44.6%	14.9%	1.04 (0.40, 2.69)
Provider perceived quality of care for obstetric complications	21.2%	18.5%	−2.7%	18.9%	36.9%	18.0%	2.24 (0.66, 7.54)

**Notes**: Except where noted, all models used generalized estimating equations with an exchangeable correlation structure. For binary quality measures, we used a log link to estimate risk ratios; for continuous measures we used the identity link. The robust sandwich estimator was used to account for clustering at the facility level and a fixed effect for district was included to account for the stratified design. The β coefficients and RR are the difference-in-differences estimates. For example, the increase in number of newborn counseling items received from baseline to endline was 0.74 items higher for women delivering in intervention facilities than women delivering in control facilities.

ANC = Antenatal care

CI = Confidence interval

^*^ *P*-value less than 0.05

^1^Difference in mean or percentage points between endline and baseline in control group (Control_endline_—Control_baseline_)

^2^Difference in mean or percentage points between endline and baseline in intervention group (Intervention_endline_—Intervention_baseline_)

^3^Composite indicator using data from facility registers. The summed proportion of deliveries where the infant was breastfed within 1 hour, the baby’s weight was recorded and a partograph was used during delivery.

^4^Composite indicator of six BEmONC signal functions reported by a senior provider to have been performed in the last 3 months: antibiotics administered parenterally, oxytocics administered perenterally, anticonvulsants administered, manual removal of the placenta, removal of retained products, newborn resuscitation.

^5^Women’s report of receipt of three services: provider checked on mother, provider checked on newborn and mother received uterotonic.

^6^Women’s report of receipt of counseling on six items: breastfeeding within the first hour of delivery, breastfeeding exclusively, care of the umbilical cord, need to avoid chilling of baby, immunization and hand washing with soap/water before touching the baby.

^7^Composite indicator of patient reported nontechnical quality. Created from ratings of provider’s explanation, respectful greeting, privacy, facility cleanliness and no disrespectful treatment (values range from 0–5). Count of those with the top rating (e.g. excellent) on Likert scale ranging from poor to excellent. No disrespectful treatment was asked as a yes/no question.

^8^Composite indicator of patient reported technical quality created from ratings of provider knowledge and availability of equipment and medications (values range from 0–2). Count of those with the top rating (e.g. excellent) on Likert scale ranging from poor to excellent.

^9^Comparison of intervention to control at endline and adjusted for age, household wealth (quintiles derived from an 18-question asset index) and district. This association is not causal and can be interpreted as the risk of not having severe anemia is the same in both intervention and control facilities at endline, after adjusting for age, household wealth and district.

^10^Quality and satisfaction questions were asked on a Likert scale from poor to excellent or very dissatisfied to very satisfied. Indicators were created to compare those with the top rating (e.g. excellent or very satisfied) to all others.

Among providers who completed the knowledge test at baseline and first follow-up, the average score in intervention facilities increased from 47.7% to 51.4%, which was not a significant increase over the change in the control facilities (Table 4).

**Table 4:** The effect of the MNH+ intervention on healthcare provider knowledge and competence, difference-in-differences analysis

	Control baseline^1^ mean	Follow-up^2^ mean	Control diff ^3^	Intervention baseline^1^ mean	Follow-up^2^ mean	Interv. diff ^4^	β (95% CI)
Change within providers							
Knowledge test^5^	0.458	0.469	0.011	0.477	0.514	0.037	0.02 (−0.04, 0.08)
Clinical vignettes^6^	0.355	0.479	0.124	0.453	0.517	0.064	−0.04 (−0.23, 0.14)
Change in facility mean score							
Knowledge test	0.459	0.490	0.031	0.462	0.516	0.054	0.02 (−0.09, 0.13)
Clinical vignettes	0.344	0.451	0.107	0.446	0.424	−0.02	−0.13 (−0.26, 0.00)

**Notes**: Difference-in-differences analysis comparing the differences in test score from baseline to endline in intervention and control arms, accounting for district. Accounting for district, the healthcare providers in the intervention facilities increased their knowledge test scores by 2 percentage points from baseline to endline above the change in test scores for providers in the control facilities.

^1^Baseline data were collected in 2011–2012

^2^The ‘follow-up’ time period varies by model. For the first model, change within providers, we used follow-up data from 2013. This was the first follow-up period and has the lowest attrition from baseline of any of the follow-up periods. For the second model, change in facility mean score, we used follow-up data from 2016, the last round of follow-up when the facility would have had the longest period of time exposed to the intervention.

^3^Difference in mean score between endline and baseline in control group (Control_endline_—Control_baseline_)

^4^Difference in mean score between endline and baseline in intervention group (Intervention_endline_—Intervention_baseline_)

^5^Trained providers (e.g. nurses and clinical officers) completed a 60-question multiple choice test on emergency obstetric and newborn care.

^6^Trained providers (e.g. nurses and clinical officers) completed two vignettes to measure provider competence on two common emergency obstetric conditions: severe preeclampsia and postpartum hemorrhage.

There was no difference in the provider knowledge test (*P* = 0.829) and vignettes (*P* = 0.306) in the years prior to and after training. Scores for individual providers increased by < 1 percentage point on the knowledge test and 3 percentage points on the clinical vignettes (*N* = 35 providers); the average follow-up time was 10.4 months. Comparatively, the administrative data from the training showed an 18.6 percentage point increase from training start to end for the same 35 individuals or 17.9 percentage points for the full cohort of 91 trained individuals.

When assessing the difference-in-differences between the high implementation facilities and the control facilities (Table 5), the only quality indicator associated with the intervention was provider obstetric knowledge (*P* = 0.40).

**Table 5:** Sub-analysis of the association between the MNH+ intervention and quality, comparing the difference in quality score from baseline to endline in the sub-group of high-implementation intervention facilities compared to the control facilities

	β (95% CI)
Structure	
Provider knowledge^1^	
Obstetric knowledge test^2^	0.05 (0.00, 0.11)
Obstetric competence vignettes^3^	−0.07 (−0.34, 0.21)
Processes	
Provision of evidence-based care	
Routine care (3 items)^4^	0.19 (−0.03, 0.40)
Basic emergency obstetric and newborn care (6 items)^5^	0.42 (−1.38, 2.21)
Receipt of services by women	
Receipt of postpartum services (3 items)^6^	−0.08 (−0.46, 0.30)
Receipt of newborn counseling (6 items)^7^	0.57 (−0.07, 1.20)
Patient experience and patient reported care competence	
Nontechnical quality^8^	0.13 (−0.06, 0.32)
Technical quality^9^	−0.10 (−0.21, 0.02)
Outcomes	
Health outcomes^10^	RR (95% CI)
Patient is not anemic	0.89 (0.76, 1.02)
Patient is not hypertensive	1.00 (0.97, 1.03)
Overall quality and satisfaction^11^	
Patient satisfaction with delivery care	0.82 (0.59, 1.13)
Patient perceived quality of delivery care	−0.01 (−0.15, 0.14)
Provider perceived quality of antenatal care	0.52 (0.13, 2.13)
Provider perceived quality of labor care	1.44 (0.41, 5.08)
Provider perceived quality of care for obstetric complications	2.02 (0.64, 6.34)

**Notes**: Difference-in-differences analysis comparing the changes from baseline to endline in high-implementation intervention facilities (N = 4) to control facilities (*N* = 12).

^1^For the provider knowledge test and provider vignettes, we analyzed the change within providers from baseline to the first follow-up in 2013. This is consistent with the main model presented in Table 4.

^2^Trained providers (e.g. nurses and clinical officers) completed a 60-question multiple choice test on emergency obstetric and newborn care.

^3^Trained providers (e.g. nurses and clinical officers) completed two vignettes to measure provider obstetric competence on two common emergency obstetric conditions: severe preeclampsia and postpartum hemorrhage.

^4^Composite indicator using data from facility registers. The summed proportion of deliveries where the infant was breastfed within 1 hour, the baby’s weight was recorded and a partograph was used during delivery.

^5^Composite indicator of six BEmONC signal functions reported by a senior provider to have been performed in the last 3 months: antibiotics administered parenterally, oxytocics administered perenterally, anticonvulsants administered, manual removal of the placenta, removal of retained products, newborn resuscitation.

^6^Women’s report of receipt of three services: provider checked on mother, provider checked on newborn and mother received uterotonic.

^7^Women’s report of receipt of counseling on six items: breastfeeding within the first hour of delivery, breastfeeding exclusively, care of the umbilical cord, need to avoid chilling of baby, immunization and hand washing with soap/water before touching the baby.

^8^Composite indicator of patient reported nontechnical quality. Created from ratings of provider’s explanation, respectful greeting, privacy, facility cleanliness and no disrespectful treatment (values range from 0 to 5). Count of those with the top rating (e.g. excellent) on Likert scale ranging from poor to excellent. No disrespectful treatment was asked as a yes/no question.

^9^Composite indicator of patient reported technical quality created from ratings of provider knowledge and availability of equipment and medications (values range from 0 to 2). Count of those with the top rating (e.g. excellent) on Likert scale ranging from poor to excellent.

^10^Comparison of intervention to control at endline and adjusted for age, household wealth (quintiles derived from an 18-question asset index) and district. This association is not causal and can be interpreted as the risk of not having severe anemia is the same in both intervention and control facilities at endline, after adjusting for age, household wealth and district.

^11^Quality and satisfaction questions were asked on a Likert scale from poor to excellent or very dissatisfied to very satisfied. Indicators were created to compare those with the top rating (e.g. excellent or very satisfied) to all others.

## Discussion

Through this cluster-randomized controlled study, we found that the quality of maternal and newborn care was low: measures of baseline quality ranged from 13 to 63% across groups. Furthermore, there was no significant improvement in the quality of care associated with the intervention; only one of the 18 metrics showed improvement. Our findings indicate issues with both implementation strength and fidelity.

Receipt of newborn counseling was the only indicator demonstrating impact by the intervention. Because newborn counseling is relatively standardized, it is cognitively easy to deliver with no additional infrastructure. It is plausible that this makes it susceptible to improvement by training and mentorship. Provider knowledge and competence, on the other hand, are heavily influenced by the providers’ abilities prior to the start of the intervention, and thus training and mentorship might be less likely to effect long-term knowledge gain and performance improvement [33, 34].

In an implementation science context, failure to effect the targeted change can be because of failure in implementation or flaws in the theory of change [21]. We found that despite an implementation manager and resources dedicated to the quality improvement project, sustaining implementation of this complex intervention was challenging. While the implementers were able to deliver equipment, supplies and medications as well as yearly trainings in BEmONC, they were not able to retain trained providers. It is likely that providers who did not receive the training early, and providers who were unskilled (e.g. medical attendants), contributed to a dilution of the effects of training [35]. The intervention was designed to be lean and thus scalable. It is possible that a more intense intervention would have led to improved outcomes; however, we found that even the high-implementation intervention facilities did not show quality improvements, suggesting that in addition to poor implementation, there were likely flaws in the theory of change.

The MNH+ intervention was built on the theory that a combination of clinic-level training, supervision, infrastructure improvement and outreach would create facility-wide improvement. However, the theory was dependent on the assumption that first level facilities are capable of improving their quality of obstetric care with an intervention targeting change at the facility level [36]. The failure of the MNH+ intervention to affect quality suggests that this theory of change may be incorrect, at least in this context of low volumes of births, weak provider skills and knowledge and poor infrastructure. Other experiences show that a theory of change can succeed in some contexts but not in others [37]. For example, after implementation of the WHO surgical safety checklist, there were improvements in quality and reduction in mortality in 8 hospitals, but a checklist adapted to maternal care in lower level clinics in India did not lead to decline in mortality or measured adverse outcomes [38, 39].

There are a number of limitations to this study. First, given that we tested 18 quality outcomes, it is possible that the one significant result—newborn counseling—was a result of statistical noise. Second, while definitions of health are often agreed on by international guidelines or norms, measures of process quality are less well defined and difficult to measure. Because all of the study facilities were low-volume primary care clinics, we were not able to conduct direct observation of care or detect significant changes in maternal mortality or morbidity. Third, given that we assess multiple outcomes that occur with different frequency and sample sizes, it is possible that small sample sizes for some of the facility-level outcomes could have contributed the inability to detect a statistically significant result. These limitations notwithstanding, it is unlikely that our approach missed substantial improvement in quality. By using numerous metrics from multiple perspectives to measure quality, we increase the strength of our conclusions: that quality is poor and was not improved by this intervention.

This study also had several strengths. The study was designed with sustainability and scalability in mind [42]. The intervention was adapted to the local context, and the intervention and research were reviewed by a local advisory committee. Finally, by nesting an in-depth evaluation of implementation within a cluster-randomized control design and developing and applying a method of implementation strength, we demonstrate rigorous methods that could translate to evaluate additional interventions.

The MNH+ intervention was carefully designed to address multiple potential levers for quality improvement; however, these were all ‘point of care’ interventions that primarily address provider behavior. Yet, the quality deficits were system-based: poor infrastructure, weak underlying provider competence and low birth volumes that precluded retaining skills. The result was little measurable change in the quality of maternal and newborn care. The limited ability of point-of-care interventions to improve quality in many low-resource contexts has been highlighted by the recent Lancet Global Health Commission on High Quality Health Systems in the Sustainable Development Goal Era [43]. Given our findings, together with these results from other recent interventions which show either minimal effect on quality and/or no effect on health outcomes after concentrated efforts to improve obstetric care quality at primary care [37, 38, 44–46], policy makers and implementers should consider testing strategies that focus on fundamental changes to the health system at higher levels, rather than the incremental, point-of-care-focused interventions that were abundant during the Millennium Development Goal era.

One potential solution is to cease efforts to improve low-volume first-level clinics and encourage childbirth at hospitals capable of providing high quality obstetric care [10], except in remote areas where distance is a major barrier or where this strategy would increase inequitable access to care. A second potential solution is based on our finding of low-level baseline knowledge of healthcare providers, suggesting a need to focus on improving pre-service education to produce a competent and ethical workforce [47]. There is a continued need for improvement in the quality of maternal and newborn care in SSA; this study provides empirical evidence of a multi-component intervention targeted at the primary care clinic that was insufficient to cause this needed change.

## Supplementary Material

Appendix_2_revised_mzz126

Appendix_3_mzz126

Appendix_4_mzz126

Appendix_5_revised_mzz126

## Appendix 1

The obstetric knowledge test was developed and administered in English, the language of medical pre-service training in Tanzania. The self-administered test included 60 multiple-choice questions that were adapted from a Jhpiego questionnaire based on international guidelines from the WHO. The test emphasized obstetric and newborn emergencies. The final provider survey consisted of two clinical vignettes. Clinical vignettes are used as an evaluation tool during medical training and increasingly used to measure quality of care of healthcare providers [48–51]. The vignettes were adapted from two Jhpiego-developed clinical vignettes for the diagnosis and management of severe pre-eclampsia and post-partum hemorrhage. Trained study nurses administered the vignettes using scripted instructions and written scenarios. Participants were instructed to respond to the scenario as if they had access to all of the resources they needed. The vignettes were written in English and translated to Swahili by the research nurses at the point of administration.
