# Supplementary material for: Did a quality improvement intervention improve quality of maternal health care? Implementation evaluation from a cluster-randomized controlled study
Source: Int J Qual Health Care. 2019 Dec 12;32(1):54–63. doi: 10.1093/intqhc/mzz126 (PMC7172021; doi:10.1093/intqhc/mzz126)
Supplement: Appendix_2_revised_mzz126 [file appendix_2_revised_mzz126.docx]

**Appendix 2a.** Summary of quality of care indicators and data collection method

| Quality of care category | Unit of analysis | Measure | Data collection tool | Frequency of data collection |
| --- | --- | --- | --- | --- |
| Structure | Provider | Obstetric knowledge | Provider knowledge test | Yearly |
| Structure | Provider | Obstetric competence | Provider competence vignettes | Yearly |
| Process | Facility | Provision of evidence-based care: routine care (summed the proportion of deliveries where the infant was breastfed within one hour, the baby’s weight was recorded, and a partograph was used during delivery) | Facility health registers | Yearly |
| Process | Facility | Provision of evidence-based care: BEmONC (antibiotics administered parenterally, oxytocics administered parenterally, anticonvulsants administered, manual removal of the placenta, removal of retained products, and newborn resuscitation) | Facility interview | Yearly |
| Process | Woman | Receipt of IV antibiotic | Household survey | Baseline, midline, endline |
| Process | Woman | Receipt of uterotonic | Household survey | Baseline, midline, endline |
| Process | Woman | Receipt of newborn counseling (breastfeeding within the first hour of delivery, breastfeeding exclusively, care of the umbilical cord, need to avoid chilling of baby, immunization, and hand washing with soap/water before touching the baby) | Household survey | Baseline, midline, endline |
| Process | Woman | Patient report of non-technical quality of care (provider’s explanation, respectful greeting, privacy, facility cleanliness, and no disrespectful treatment) | Household survey | Baseline, midline, endline |
| Process | Woman | Patient report of technical quality of care (provider knowledge and availability of equipment and medications) | Household survey | Baseline, midline, endline |
| Outcome | Woman | Patient is not anemic | Household survey | Midline, endline |
| Outcome | Woman | Patient is not hypertensive | Household survey | Midline, endline |
| Outcome | Woman | Maternal mid-upper arm circumference | Household survey | Baseline, midline, endline |
| Outcome | Woman | EQ-5D | Household survey | Baseline, midline, endline |
| Outcome | Woman | Patient satisfaction with delivery care | Household survey | Baseline, midline, endline |
| Outcome | Woman | Patient perceived quality of delivery care | Household survey | Baseline, midline, endline |
| Outcome | Provider | Provider perceived quality of ANC | Provider interview | Yearly |
| Outcome | Provider | Provider perceived quality of labor care | Provider interview | Yearly |
| Outcome | Provider | Provider perceived quality of care for obstetric complications | Provider interview | Yearly |

**Appendix 2b.** Data collection methods to construct a single composite measure of implementation strength

Data were collected from five separate data collection tools outlined in Table 2. The supportive supervision checklists and interviews were completed using handheld tablets with ODK-based software. Data collection details for the register data and facility assessments have been previously reported in detail.

| **Data collection tool** | **Overview of data collected** | **Person completing tool** | **Dates of data collection** |
| --- | --- | --- | --- |
| Implementation activity log | Date and location of supportive supervision visits; date and type of equipment, supplies, and medications delivered; date and type of infrastructure renovations | Implementation manager | Completed weekly from June 2012-June 2016 |
| Training report | Description of training, number and type of providers trained, provider training scores | Implementation manager | July 2012, November 2013, August 2015 |
| Supportive supervision checklist | Detailed description of activities conducted during visit and supervisors and supervisees present | Implementation manager | Completed at each visit from January 2013-May 2016 |
| Facility assessment | Number and type of providers; facility catchment area; availability of essential MNH equipment, supplies, and drugs; impression of last supportive supervision visit | Independent research assistants | December 2011-May 2012; February 2013; February-March 2014; June 2015; January-February 2016 |
| Facility record abstraction | Number of visits to facility by outside organizations; number of deliveries conducted; number of outpatient visits conducted | Independent research assistants | Monthly aggregate data from January 2012-December 2015 |

Construction of the implementation index:

$${\sum_{c=1}^{n} (D_{c}+R_{c}+E_{c})}/n$$

**Notes**: c is each component of the intervention. In this case n is three (infrastructure, training, and supportive supervision/mentorship). D is the indicator for dose delivered, R is the indicator for reach, and E is the indicator for dose received.
