# Supplementary material for: Did a quality improvement intervention improve quality of maternal health care? Implementation evaluation from a cluster-randomized controlled study
Source: Int J Qual Health Care. 2019 Dec 12;32(1):54–63. doi: 10.1093/intqhc/mzz126 (PMC7172021; doi:10.1093/intqhc/mzz126)
Supplement: Appendix_3_mzz126 [file appendix_3_mzz126.docx]

**Appendix 3.** Study eligibility and analysis flow diagram for providers and patients.

Notes: Data on the number of providers eligible were collected during the yearly facility assessments.

a The number of eligible providers was listed as two fewer than the number interviewed; eligible providers adjusted to reflect the number interviewed

b Of the 76 facilities that were 1) located in a study district, 2) primary care clinics (dispensary), and 3) supported by Tanzania Health Promotion Support, 6 were excluded because they were private and 2 were exclude because they had a large maternal and newborn health quality improvement intervention ongoing. From the remaining 68 facilities we selected the six with the most deliveries. If one of the selected facilities did not have a skilled provider (n=3) we selected the next eligible facility with the most deliveries.
