# Supplementary material for: Did a quality improvement intervention improve quality of maternal health care? Implementation evaluation from a cluster-randomized controlled study
Source: Int J Qual Health Care. 2019 Dec 12;32(1):54–63. doi: 10.1093/intqhc/mzz126 (PMC7172021; doi:10.1093/intqhc/mzz126)
Supplement: Appendix_4_mzz126 [file appendix_4_mzz126.docx]

**Appendix 4.** Provider characteristics for those who participated in at least one of the surveys in that study year.

|  | **2012** |  | **2013** |  | **2014** |  | **2015** |  | **2016** |  |  |
| --- | --- | --- | --- | --- | --- | --- | --- | --- | --- | --- | --- |
|  | **Control** | **Intervention** | **Control** | **Intervention** | **Control** | **Intervention** | **Control** | **Intervention** | **Control** | **Intervention** |  |
| Respondents | (N=35) | (N=51) | (N=48) | (N=48) | (N=50) | (N=61) | (N=40) | (N=64) | (N=54) | (N=65) |  |
| Female | 27 (77.1) | 38 (74.5) | 37 (77.1) | 38 (79.2) | 40 (80) | 48 (78.7) | 28 (70) | 48 (75) | 38 (70.4) | 47 (72.3) |  |
| Age (mean, SD) | 42.3 (9.0) | 37.9 (9.7) | 40.4 (10.0) | 36.2 (10.1) | 36.4 (10.7) | 35.6 (9.8) | 34.8 (9.9) | 31.6 (10.3) | 35.5 (10.4) | 33.0 (10.8) |  |
| Cadre |  |  |  |  |  |  |  |  |  |  |  |
| Clinical officer | 12 (34.3) | 14 (27.5) | 15 (31.2) | 15 (31.2) | 12 (24) | 18 (29.5) | 8 (20) | 17 (26.6) | 11 (20.4) | 14 (21.5) |  |
| Nurse | 8 (22.9) | 10 (19.6) | 8 (16.7) | 11 (22.9) | 12 (24) | 18 (29.5) | 11 (27.5) | 19 (29.7) | 17 (31.5) | 21 (32.3) |  |
| Medical attendant Φ | 14 (40) | 24 (47.1) | 22 (45.8) | 21 (43.8) | 22 (44) | 23 (37.7) | 18 (45) | 23 (35.9) | 22 (40.7) | 25 (38.5) |  |
| Other | 1 (2.9) | 3 (5.9) | 3 (6.2) | 1 (2.1) | 4 (8) | 2 (3.3) | 3 (7.5) | 5 (7.8) | 4 (7.4) | 5 (7.7) |  |
| Full time employment | 30 (90.9) | 36 (97.3) | 45 (93.8) | 44 (91.7) | 49 (98) | 56 (98.2) | 37 (94.9) | 55 (100) | 52 (96.3) | 64 (98.5) |  |
| Worked in study facility for more than 2 years | 29 (87.9) | 22 (59.5) | 37 (77.1) | 32 (66.7) | 34 (68) | 40 (70.2) | 40 (70.2) | 28 (71.8) | 37 (68.5) | 36 (55.4) |  |
| District of employment |  |  |  |  |  |  |  |  |  |  |  |
| Bagamoyo | 11 (31.4) | 13 (25.5) | 11 (22.9) | 16 (33.3) | 12 (24) | 19 (31.1) | 10 (25) | 10 (15.6) | 17 (31.5) | 18 (27.7) |  |
| Kibaha Rural | 10 (28.6) | 11 (21.6) | 12 (25) | 10 (20.8) | 13 (26) | 14 (23) | 8 (20) | 21 (32.8) | 13 (24.1) | 12 (18.5) |  |
| Kisarawe | 10 (28.6) | 15 (29.4) | 16 (33.3) | 16 (33.3) | 15 (30) | 18 (29.5) | 9 (22.5) | 16 (25) | 14 (25.9) | 20 (30.8) |  |
| Mkuranga | 4 (11.4) | 12 (23.5) | 9 (18.8) | 6 (12.5) | 10 (20) | 10 (16.4) | 40 (100) | 64 (100) | 10 (18.5) | 15 (23.1) |  |
| **Facility characteristics** |  |  |  |  |  |  |  |  |  |  |  |
| Workload^ |  |  |  |  |  |  |  |  |  |  |  |
| Number of facility deliveries (mean, SD) | 5.9 (3.8) | 7.9 (6.7) | 11.4 (9.4) | 10.7 (8.5) | 13.4 (10.4) | 10.9 (8.6) | 14.2 (10.8) | 12.3 (11.0) | 12.7 (10.8) | 9.0 (8.4) |  |
| Number of outpatient visits (mean, SD) | 240.1 (149.6) | 255.4 (143.9) | 260.9 (123.1) | 259.5 (174.3) | 279.4 (132.4) | 254.8 (143.6) | 316.1 (120.5) | 328.9 (144.8) | 327.0 (132.0) | 354.8 (159.2) |  |
| Number of healthcare workers at facility (mean, SD) | 3.6 (1.4) | 4.2 (1.4) | 4.2 (1.8) | 5.1 (2.0) | 4.8 (2.3) | 5.6 (2.2) | 4.8 (1.9) | 4.6 (2.1) | 4.8 (1.4) | 5.0 (2.5) |  |

Numbers may not add up to total as a result of missing values.

Φ Includes medical attendants and maternal and child health aides.

^ “2012” data represent utilization from January – December 2011; “2013” data represent utilization from January – December 2012; “2014” data represent utilization from January – December 2013; “2015” data represent utilization from January – December 2014; “2016” data represent utilization from January – December 2015
