# Supplementary material for: Did a quality improvement intervention improve quality of maternal health care? Implementation evaluation from a cluster-randomized controlled study
Source: Int J Qual Health Care. 2019 Dec 12;32(1):54–63. doi: 10.1093/intqhc/mzz126 (PMC7172021; doi:10.1093/intqhc/mzz126)
Supplement: Appendix_5_revised_mzz126 [file appendix_5_revised_mzz126.docx]

**Appendix 5.** The effect of the MNH+ intervention on the quality of care in government-managed primary healthcare from 2012 to 2014 (midline), difference-in-differences analysis

|  |  | β (95% CI) |  |
| --- | --- | --- | --- |
| Processes | |  |  |
| Provision of evidence-based care | |  |  |
|  | Routine care (3 items)^1^ | 0.26 (0.10, 0.42) |  |
|  | Basic emergency obstetric and newborn care (6 items)^2^ | 0.58 (-0.92, 2.09) |  |
| Receipt of services by women | |  |  |
|  | Receipt of postpartum services (3 items)^3^ | 0.18 (-0.01, 0.37) |  |
|  | Receipt of newborn counseling (6 items)^4^ | 0.33 (-0.13, 0.79) |  |
| Patient experience and patient reported care competence | |  |  |
|  | Non-technical quality^5^ | -0.03 (-0.33, 0.27) |  |
|  | Technical quality^6^ | -0.03 (-0.17, 0.11) |  |
| Outcomes | |  |  |
| Health outcomes^7^ | | RR (95% CI) |  |
|  | Patient is not anemic | 0.94 (0.79, 1.13) |  |
|  | Patient is not hypertensive | 1.03 (0.98, 1.07) |  |
| Overall quality and satisfaction^8^ | |  |  |
|  | Patient satisfaction with delivery care | 1.04 (0.76, 1.40) |  |
|  | Patient perceived quality of delivery care | 0.80 (0.41, 1.56) |  |
|  | Provider perceived quality of antenatal care | 1.02 (0.27, 3.84) |  |
|  | Provider perceived quality of labor care | 1.37 (0.52, 3.60) |  |
|  | Provider perceived quality of care for obstetric complications | 1.75 (0.58, 5.23) |  |

**Notes**: Difference-in-differences analysis comparing the changes from baseline to endline in high-implementation intervention facilities (implementation index > 33%, N=5) to control facilities (N= 12).

1 Composite indicator using data from facility registers. The summed proportion of deliveries where the infant was breastfed within one hour, the baby’s weight was recorded, and a partograph was used during delivery. Data from midline are from January-December 2013.

2 Composite indicator of six BEMONC signal functions reported by a senior provider to have been performed in the last three months: antibiotics administered parenterally, oxytocics administered perenterally, anticonvulsants administered, manual removal of the placenta, removal of retained products, newborn resuscitation.

3 Women’s report of receipt of three services: provider checked on mother, provider checked on newborn, and mother received uterotonic.

4 Women’s report of receipt of counseling on six items: breastfeeding within the first hour of delivery, breastfeeding exclusively, care of the umbilical cord, need to avoid chilling of baby, immunization, and hand washing with soap/water before touching the baby.

5 Composite indicator of patient reported non-technical quality. Created from ratings of provider’s explanation, respectful greeting, privacy, facility cleanliness, and no disrespectful treatment (values range from 0-5). Count of those with the top rating (e.g. excellent) on Likert scale ranging from poor to excellent. No disrespectful treatment was asked as a yes/no question.

6 Composite indicator of patient reported technical quality created from ratings of provider knowledge and availability of equipment and medications (values range from 0-2). Count of those with the top rating (e.g. excellent) on Likert scale ranging from poor to excellent.

7 Comparison of intervention to control at endline and adjusted for age, household wealth (quintiles derived from an 18-question asset index), and district. This association is not causal and can be interpreted as the risk of not having severe anemia is the same in both intervention and control facilities at endline, after adjusting for age, household wealth, and district.

8 Quality and satisfaction questions were asked on a Likert scale from poor to excellent or very dissatisfied to very satisfied. Indicators were created to compare those with the top rating (e.g. excellent or very satisfied) to all others
